# Is Prosthetic Dentistry Lagging?

**Published:** 1894-12

**Authors:** William H. Trueman

**Affiliations:** Philadelphia


### IS PROSTHETIC DENTISTRY LAGGING? NO.[1]

BY WILLIAM H. TRUEMAN, D.D.S., PHILADELPHIA.

"WHILE the reputation of the dental profession in general has been gradually advancing in regard to its knowledge of therapeutics, and in the treatment of oral diseases, together with a still greater advancement in all operations upon the natural teeth, there has not been a corresponding progress in prosthetic dentistry."

---

[1] Read at a meeting of the Pennsylvania Association of Dental Surgeons, October 9, 1894.

This statement in the first paragraph of Dr. Broomell's paper, in the October (1894) number of the INTERNATIONAL DENTAL JOURNAL, on "Metallic *versus* the Plastic Base for Artificial Dentures," suggests to my mind the above question, and challenges a reply. Confining my remarks closely to this one question, I do not propose to criticise the paper further than to say that it lacks throughout that thorough study and thoughtful sifting of evidence through which scientific conclusions should always be sought. I can justly add, however, that it does so to no greater degree than do the vast majority of articles that make up the general literature of to-day.

We speak of the progress of dental science, and of the relative progress of operative and prosthetic dentistry. Now, let us first fix in our minds what we mean by these expressions. I take it (in brief) that the end and aim of those who practise dentistry is, as far as in their power lies with the means and material at command, to lessen the discomforts suffered by the community in which they live and work, due to the presence or to the absence of the natural teeth. I take it that the progress of dental science is to be judged by its ability to make happier and more pleasant the life of each individual of the community in so far as their pleasure and happiness can be enhanced by dental art. Regarding relative progress, it seems reasonable to assume that the nearer that branch of dentistry chiefly concerned in replacing with artificial substitutes the lost natural teeth approaches perfection,—perfection being that state wherein the artificial fully supplants the natural,—in a corresponding degree will the preservation of the natural teeth become of lessened importance. If it were possible to enjoy the same comfort with a mouth full of artificial teeth, teeth that never decay, never ache, that can be taken out for the purpose of cleansing, and, in case of accident, can be boxed up and sent away for repairs, their place in the mean time being occupied by a duplicate set, who would for a moment longer than absolutely necessary be bothered by the discomforts and ills, and the continual expense of time and money so many sets of natural teeth entail upon their owners, from the moment they appear until they are finally lost?

I presume, however, that nothing is risked in saying that the most skilful practitioner of prosthetic dentistry has not yet reached that point; and that there is much to be done before the ideal perfect set of natural teeth, and the best substitutes our science can produce, will be considered interchangeable. Nevertheless, it is indisputable, there is no question on this point; many natural teeth do not, in their character and structure, in their ability to resist the destructive tendencies of their surroundings, and in their relation to the general system, closely approach the ideal; indeed, many so far fall short of it that, notwithstanding all that the most advanced in the science of tooth preservation have so far been able to accomplish, there are cases where their loss and replacement by artificial substitutes has materially added to the patient's comfort and well-being. It is also beyond question that such cases are fewer in number to-day than they were half a century ago, and we may reasonably assume that they will be still less numerous half a century hence. All this, however, is beside the question at issue. It simply shows that operative dentistry, while it has not yet reached its ideal, is more and more successfully accomplishing its mission of preserving in comfortable usefulness the natural organs.

Now, then, to the real issue. In the first place, is the dentist of to-day as a mechanical workman less able? Is he less successful in supplying his patients with artificial dentures? Are the artificial teeth of the present time worn with less comfort and satisfaction than were those of—— Where shall we fix the date for comparison? Accepting the suggestion of Dr. Broomell, let us say, since the advent of vulcanite. There seems to be an impression among many who speak and write upon this subject that, before the advent of vulcanite, all dentists were experts in the manipulation of the metallic bases which it supplanted. I can imagine nothing further from the truth.

Although there were at that time many skilful workmen who did beautiful work, the average work done was, in my judgment, poor, and to the patient far less useful and comfortable than that of to-day. Dr. Broomell's suggestion that "vulcanized rubber is the nucleus from which sprang dental quackery" is absurd,—amusingly so to a better informed but thoughtless man. To me such expressions are saddening, they are humiliating, they indicate the utter absence of thoughtful preparation among those who pose as exponents of dental thought. Writers of a century and a half ago speak as glibly of dental quacks, and empirical pretenders who cut prices, as do some writers of the present time. Such expressions are saddening in that they most forcibly bring home to us the fact that this calling of ours, masquerading year after year as a liberally learned profession, has utterly failed to provide for its studiously-inclined members such facilities for study which other professions find so necessary and provide so bountifully. Is it possible, can it be true, that there is not in the whole United States, outside of private ownership, *a single dental library worthy of the name?*

I wonder if the author of that expression has ever seen a whole set of artificial teeth on gold or silver base, the plates made to conform to the plaster cast by bending to shape with the pliers and the skilful use of the hammer and bench-anvil, perfectly innocent of any contact with die or counter-die; single gum teeth arranged in place without a touch of the grindstone, and the case, after a rough soldering, passed from the pickel-pot to the mouth with but a few minutes work in the way of smoothing and polishing with a coarse file and sand-paper. I have consigned many such cases to the melting-pot. Talk of filthy vulcanite, more than once, when a laboratory workman, I have been compelled, before handling them, owing to their extreme filthiness, to pass gold or silver repair cases from the box in which they came into a disinfectant, and immediately after, overcome by the noisome odor, have emptied the meal last eaten into the laboratory sink. The condition of the mouths from which those plates came I leave you to imagine.

I presume it is true that the average dentist of to-day is not so well versed as was the average dentist of the fifties and sixties in the manipulation of gold and silver. He may not be able to refine gold and silver, cast it into ingots, and form it into plate. He would probably be completely nonplussed if required to reduce the ingot to plate by forging with hammer on anvil, yet that was the way in which one of my laboratory predecessors *always* made his plates; in his judgment, the rolling-mill was a makeshift for the lazy or the unskilful. By forging alone he produced a plate almost as smooth and of as even thickness as though it had been rolled. The average dentist of to-day may not be able to do as did Dr. Thomas Evans, of Paris, who within a few hours, at night, with the meagre appliances of a country jeweller's shop, transformed a five-franc piece into a silver canula, and thereby prolonged the life of the late German emperor. Dr. Maynard made all his instruments with his own hands except the forging of his extracting forceps, and pitied the dentist unable to do the same. Skilled operators of an earlier date beat out their "joes"[1] with their own hands, and from them made all the gold-foil they used in filling. How many operators of to-day are capable of doing either? Is their inability in these lines any evidence of a degeneracy?

Why should the prosthetic dentist of to-day waste time in ac-

---

[1] Joe, a short name commonly used for Johanne, a Portuguese gold coin, value about $8.64, made of almost pure gold, and on that account preferred by the dentists of that time for making foil.

quiring the art of manipulating gold and silver, an art difficult to learn, and retained only by constant practice, when he feels he can do much better for his patients by using vulcanite? I know thoroughly well all the objections that have been urged against it. I helped to make the record of its vices, but am now free to admit, as was the case with many another so it was with me; when the patent expired and license for its use was no longer required, its many vices dwarfed and its virtues grew. I refer you to an interesting discussion on the subject, reported in the *Dental Cosmos*, vol. vi. (1869), page 80. Much that was there said is as true to-day as it was a quarter of a century ago, and much is very like the stuff found in reports of a more recent date.

Oh, yes, I know all about the ill-made joints of the modern vulcanite plate, the occasional porous rubber, and the rough places that should have been made smooth. Yes, it is true, I admit it, that with some (we will say with many, if you prefer it so) the easy work of making vulcanite plates has engendered careless habits. I admit that the poorly-made vulcanite plate furnishes many a choice spot in which microbes live, increase, grow fat, and run riot; it is equally true that in many gold and silver dentures of a little more than thirty years ago, not only were the joints more numerous, but too often between the teeth and the plate there existed a tunnel through which, with a little "hunching," a cockroach might crawl. Is it backward or forward, this step from cockroaches to microbes?

It is true, the easy-working rubber has given a distaste for the long, tedious, exacting processes by which the skilful workman produced the exquisite artistic finish seen in some of the gold and silver dentures of the past. Many a long and weary day have I spent in rubbing, rubbing, rubbing with Scotch stone and pumice, surfacing gold or silver plates until not a scratch or a dimple could be seen with a watch maker's glass, and then some two hours or more of pumice and oil, dry rotten-stone, and, finally, rough with brush-wheels at the polishing lathe. After the graver had done its work, a full day was considered none too long a time to finish a full upper or lower denture. Never again will I spend so long a time in such tedious work, until it is the fashion to wear such dentures inside out. So far as practical utility is concerned, the modern felt-wheel will do the work quite as well in one-tenth the time. The finish may not be so artistic, but what of that? It is never seen when in position in the patient's mouth, where it belongs, and the time can be better spent in something more useful. The advent of bridge-work has proved beyond question that there still exists in the profession quite as much mechanical ability and of as high an order as there ever did. The exquisite work in the plastic bases, artistic and mechanical, exhibited at dental meetings, and so often seen doing efficient work in patients' mouths, testifies alike to the ability of the prosthetic dentist who constructed it, and the capability of the material of which it is made, to respond effectively to well-directed efforts. Were the plastic bases swept out of existence, and the prosthetic dentist of to-day compelled to use the materials available before their introduction, I have no question but that as good work, and as much good work, would be done now as was done then. Such expressions as have called forth this paper are among the stock "fads" constantly used in dental meetings, expressions learned parrot-like one from another, none taking the trouble to examine whether there is anything back of them or not.

That the advent of vulcanite has made less costly artificial dentures, that it has enabled a dentist possessing but little mechanical skill to construct dentures that fit the mouth better, and give the patient more satisfaction than would dentures made of a less manageable material by the same hands, is no evidence of a decline in the prosthetic dental art. This is perfectly in line with that which we term *progress* in other arts and sciences. The substitution of machinery for hand-labor, improved processes, etc., has cheapened food and clothing, has resulted in the community being better fed, better clothed, and more comfortably housed. Many luxuries that a few years ago the wealthy alone could afford have by these means been so reduced in cost that they have become not simply accessible, but so common that they are now recognized necessities among those of limited means. Is it not reasonable to expect that, in time, dentistry, in all its branches, will be added to them? All this, while the producers of these things have easier work, fewer hours of labor, and in many cases are better paid. For from five to eight or ten dollars it is possible to obtain an upper denture on vulcanite far superior in every way to one that would have cost, perhaps, twice as much before vulcanite was known. This has enabled thousands to enjoy the comfort and satisfaction of artificial dentures who thirty or forty years ago would have been constrained, on account of their limited means, to do without them? That it has caused a less value to be set upon the natural teeth by many is also true; but do you not see in this evidence that the prosthetic branch in its service to the community has advanced beyond the operative? Take an average set of natural teeth, such as we are called upon daily to treat in the mouth of a patient, to whom a dollar saved from actual living expenses means many hours of toil; as operative dentistry is now practised, how far will five, eight, or ten dollars go towards saving those teeth? What is operative dentistry doing to-day towards adapting its methods to meet such cases?[1] That at the Columbian Dental Congress the report of the Committee on the Care of the Teeth of the Poor was read by *title only*, shows very plainly the little interest the profession takes in this important matter. It is an issue, however, that operative dentistry must meet, or fail in its mission. The increased attention given to the plastics, the improvements in material, and in methods of its manipulation, have been steps in the right direction. Dr. W. D. Miller's recent investigations with a view to simplify and render less expensive the treatment of pulp lesions is another step that deserves all the encouragement that can be given to it. The wholesale destruction of good natural teeth is largely due to inability of a vast majority of the community to command or to pay for any other attention. At the present time, as in the past, if we may judge from the reports and papers found in the dental journals, the profession discourages any attempt to meet this condition, stigmatizing as "quacks" those who are willing to work for a price the poor are willing and able to pay, grudging even the little charity work done in the college clinics.

Notwithstanding the prattle heard in our dental gatherings to the contrary, dentistry—prosthetic and operative—is a part and parcel of the world's business, and is governed by the same natural laws that governs every other industrial calling. Over thirty years of practical use has demonstrated vulcanite to be a good base for artificial dentures; its use is world-wide, yet complaints of its evil effects are far less frequent now than they were twenty years ago. Competition has reduced the cost and improved the quality of artificial dentures so that to-day few, if any, for financial reasons, need to do without them. Operative dentistry still remains a luxury the well-to-do alone can afford, and if service to the community be the test, it is not prosthetic dentistry that is lagging in the race.

Operative dentistry so far caters only to the wealthy; expensive methods, expensive materials, alone seems to attract attention. We are urged by one to use more scientific methods, *and charge for them;* by another, to do the very best we can regardless of expense and then *make the patients pay.* You will not have to search far

---

[1] Dental Cosmos, vol. xxxv. (1893), page 1051.

among reports of dental meetings to find such like expressions repeatedly used. Would it not be well to have a little thought for those "precious organs" in the mouths of the hewers of wood and drawers of water. At least have a kindly feeling for those fellow-workers, who amid great discouragements are doing their best to teach these poor work-folks how precious they are. Their fees are small, and there is often a long wait between earning and getting; indeed, too often the getting never comes. The dentist who is striving to do his duty with a practice largely composed of working people deserves far more encouragement from his fellows than he usually gets. If you cannot help him, if you cannot point him to better ways that are to him practical, don't ostracize him, don't call him vile names,—a quack, a scalawag, or a scab. Remember he is working for God's poor.

